# Risk Stratification and Coronary Optical Coherence Tomography Findings in Asymptomatic Patients With Type 1 Diabetes Mellitus

**DOI:** 10.33549/physiolres.935614

**Published:** 2025-10-01

**Authors:** Robert ROLAND, Michal DUBSKY, Peter WOHLFAHRT, Natalia MARHEFKOVA, Zhi CHEN, Milan SONKA, Vladimir KARMAZIN, Martin HALUZIK, Josef KAUTZNER, Michal PAZDERNIK

**Affiliations:** 1 Department of Cardiology, Institute of Clinical and Experimental Medicine, Prague, Czech Republic; 2 Second Faculty of Medicine, Charles University, Prague, Czech Republic; 3 Diabetology Centre, Institute of Clinical and Experimental Medicine, Prague, Czech Republic; 4 Preventive Cardiology Centre, Institute of Clinical and Experimental Medicine, Prague, Czech Republic; 5 Iowa Institute for Biomedical Imaging, The University of Iowa, Iowa City, Iowa, USA; 6 Cardiology Department, Second Faculty of Medicine, Charles University and Motol University Hospital, Prague, Czech Republic

**Keywords:** Type 1 diabetes mellitus, Coronary artery calcium score, Carotid ultrasound, Optical coherence tomography, Cardiovascular risk

## Abstract

Regarding cardiovascular (CV) risk, patients with type 1 diabetes mellitus (T1D) are a heterogeneous population with CV risk ranging from low to very high. For personalized prevention strategies, screening for subclinical atherosclerosis may be of clinical significance. However, more data is needed. Our study aimed to describe the prevalence of prognostically significant findings on invasive coronary artery examination in patients with subclinical atherosclerosis determined by non-invasive examination of the carotid and coronary arteries. Patients with T1D for at least 10 years, without a prior history of atherosclerotic CV disease or target organ damage, followed at a large tertiary hospital were enrolled. Non-invasive examinations included carotid ultrasound for carotid plaque detection and a CT for coronary artery calcium (CAC) score evaluation. Patients with the presence of ≥2 carotid plaques and/or CAC score of ≥400 were classified as very high risk (VHR). These VHR patients were subsequently evaluated using invasive coronary angiography (ICA) for the presence of obstructive coronary artery disease (CAD) and intracoronary optical coherence tomography (OCT) for the presence of thin-cap fibroatheroma (TCFA) and very high-risk plaque. Moreover, hemodynamic stenosis relevance was assessed by the vessel fraction flow ratio (vFFR). Sixty-two T1D patients aged 50.1±12.7 years, 53 % women were enrolled. The criteria of VHR were fulfilled in 12/62 (19.4 %) patients, of which 6 (50 % of VHR) had both CAC≥400 and at least 2 atherosclerotic plaques in the carotid arteries, one patient (8 % of the VHR) fulfilled only the CAC criteria and 5 (42 % of VHR) only the carotid criteria. The median CAC score of the VHR group was 606.3 (175.3–1515) and the mean number of carotid plaques was 2.75±1.06. ICA showed obstructive CAD in 5/12 (41.7 %) patients, and 3/12 (25 %) had vFFR-positive lesions. Using OCT, TCFA was present in 7/12 (58.3 %) and a very high-risk plaque in 4/12 (33.3 %) patients. Among asymptomatic patients with T1D, the combination of coronary artery calcium score and carotid ultrasound identifies a very high-risk group, in which 58.3 % of patients had a thin-cap fibroatheroma and 33.3 % of patients had a very high-risk plaque. Patients identified by these non-invasive techniques may benefit from intensive risk factors management.

## Introduction

Diabetes mellitus (DM) is one of the major independent, causal, and modifiable risk factors for atherosclerotic cardiovascular disease (CVD) [1]. Patients with type 1 diabetes (T1D) are a frequently overlooked group of DM patients, as T1D accounts for only 5–10 % of diagnosed cases. On the other hand, due to an earlier onset of T1D in life and three to four extra decades of hyperglycemia, T1D are at even higher risk of CVD, as compared to those with type 2 diabetes (T2D). T1D patients present with coronary artery disease (CAD) at a younger age and have more complex, diffuse, and peripheral atherosclerotic changes in coronary arteries. Because cardiovascular risk in T1D may vary from low to very high, risk stratification is important to personalize the preventive measures. For risk stratification, non-invasive imaging modalities such as coronary artery calcium (CAC) score or carotid ultrasound may be important. However, there is a lack of data on the clinical utility of these methods in T1D patients.

Recent advances in invasive coronary imaging using optical coherence tomography (OCT) have allowed us to visualize the thin-cap fibroatheromas (TCFA), which cannot be detected by invasive coronary angiography (ICA) or CAC score due to echo-lucent properties. Patients with TCFA are at increased risk of major adverse cardiovascular events (MACE) [2]. Recently, the PREVENT trial has demonstrated that percutaneous coronary intervention (PCI) of non-stenotic TCFA decreases cardiovascular risk [3]. This finding further supports the clinical importance of identifying patients with vulnerable plaque presence. However, no previous study has described the prevalence of TCFA among patients with T1D and subclinical atherosclerosis presence on non-invasive imaging tests.

Thus, the present study aimed to describe the prevalence of prognostically significant findings on invasive coronary artery examination (TCFA, high-risk plaque) in T1D patients with subclinical atherosclerosis determined by non-invasive examination on the carotid and coronary arteries.

## Methods

Between February and July 2023, patients with T1D followed at the Institute of Clinical and Experimental Medicine (IKEM), Prague, Czech Republic, were prospectively enrolled. The inclusion criteria were defined according to the European Society of Cardiology Guidelines on Cardiovascular Disease Prevention definition of the high CVD risk categories [4]. Patients aged ≥30 years with T1D duration of at least 10 years, who have been followed at the Diabetes Center IKEM were included and defined as a high risk (HR) group. Exclusion criteria were: symptoms of coronary artery disease (chest pain, dyspnea, peripheral edema, syncope), prior history of atherosclerotic cardiovascular (CV) disease or target organ damage (estimated glomerular filtration rate (eGFR) of <60 ml/min/1.73 m^2^, albumin-to-creatinine ratio of >30 mg/g or presence of microvascular disease in at least three different sites (e.g. microalbuminuria plus retinopathy plus neuropathy).

All recruited patients signed a written informed consent. The study protocol was approved by an Ethics Committee of IKEM and Thomayer’s Hospital. The study was conducted in accordance with all applicable regulatory requirements, the International Council for Harmonization Good Clinical Practice guidelines, and principles that have their origin in the Declaration of Helsinki.

### Non-invasive examination

Carotid ultrasound for carotid plaques and CAC score was performed in all recruited individuals = HR patients. Carotid ultrasound was performed on the Philips Affiniti 70G device. Plaque was defined as a focal structure that demonstrates a thickness ≥1.5 mm as measured from the media-adventitia interface to the intima-lumen interface [5]. CAC score was performed on the computer tomography scan (Naeotom Alpha; Siemens Healthineers, Germany) and evaluated using a dedicated software (CaScoring, Syngo.Via VB60; Siemens Healthineers, Germany). The total CAC score was calculated based on the Agatston method [6].

### Invasive examination

ICA and OCT were indicated only in very high risk (VHR) patients defined as having – 1) presence of ≥2 carotid plaques of ≥1.5 mm thickness and/or 2) CAC score of >400.

#### Invasive coronary angiography

ICA evaluated the presence of obstructive coronary artery disease (defined as a presence of ≥1 stenosis of ≥50 % in the vessel of ≥2.5 mm diameter) in all three major coronary vessels. Coronary angiography was performed *via* the transradial approach using a 6F sheath.

#### Functional stenosis assessment

To assess functional stenosis relevance derived from the coronary angiogram, a vessel fractional flow reserve (CAAS-vFFR, Pie Medical Imaging, Maastricht, the Netherlands) of all three major coronary vessels was performed following the invasive examination. vFFR is a validated method based on the 3D quantitative coronary angiography (QCA), simplified fluid equations and has a good linear correlation with wire-derived fraction flow reserve (FFR) with the same positive cut-off value of ≤0.80 [7].

#### Optical coherence tomography

OCT examination was performed using a commercially available intracoronary frequency-domain OCT system (Ilumien/Dragonfly Optis; Abott, USA). The OCT imaging catheter was advanced to each of the three major coronary vessels. Imaging of the 54 mm proximal segment at the rate of 18 mm/s was initiated automatically after blood clearing with 10 to 14 ml contrast media. The inability to obtain pullback in one of the coronary arteries was caused by small vessel diameter, vessel angulation, or severe spasm of the radial artery.

#### OCT imaging and analysis

Definitions of OCT image characteristics were based mainly on previously published international consensus [8]. The OCT analysis of plaque composition was performed manually. Each individual plaque type was accounted as present or non-present in the examined vessel. In case of several morphology, all were labeled as present. Lipid plaque was defined as a signal-poor region with a poorly defined or diffuse border, and the degree of lipid arc was measured in lipid plaques. Fibrous cap thickness (FCT) overlying a lipid plaque was measured manually 3 times at its thinnest part, and the average value was calculated. Lipid-rich plaque was defined as a plaque with a maximal lipid arc >90°. Thin-cap fibroatheroma (TCFA) was defined as a plaque with maximal lipid arc >90° and thinnest fibrous cap thickness ≤65 μm. Macrophage accumulation was defined as the presence of highly backscattering focal regions within the fibrous cap. Neovascularization (microvessels) was defined as the presence of signal-poor structures with vesicular or tubular shapes. Cholesterol crystal was identified as a thin and linear region of high signal intensity with high backscattering within a plaque. Calcification was defined as a signal-poor or heterogeneous region with sharply delineated borders. Spotty calcium was defined as the presence of a lesion containing calcification arc <90° and extending in length for 1 to 4 mm. Thrombus was defined as an irregular mass with a minimum diameter >250 μm adherent to the vessel wall or floating within the lumen. Plaque erosion was identified by the presence of either an attached thrombus overlying an intact and visualized plaque, a luminal surface irregularity in the absence of thrombus, or attenuation of underlying plaque by thrombus without superficial lipid or calcification immediately proximal or distal to the site of thrombus.

#### Primary and secondary imaging endpoints

Two primary imaging endpoints associated with a high risk of MACE were chosen [2,9]. The presence of TCFA and a very high-risk plaque defined as TCFA phenotype with >180° lipid arc, presence of macrophages, and minimal lumen area <3.5 mm^2^.

**Fig. 1:**
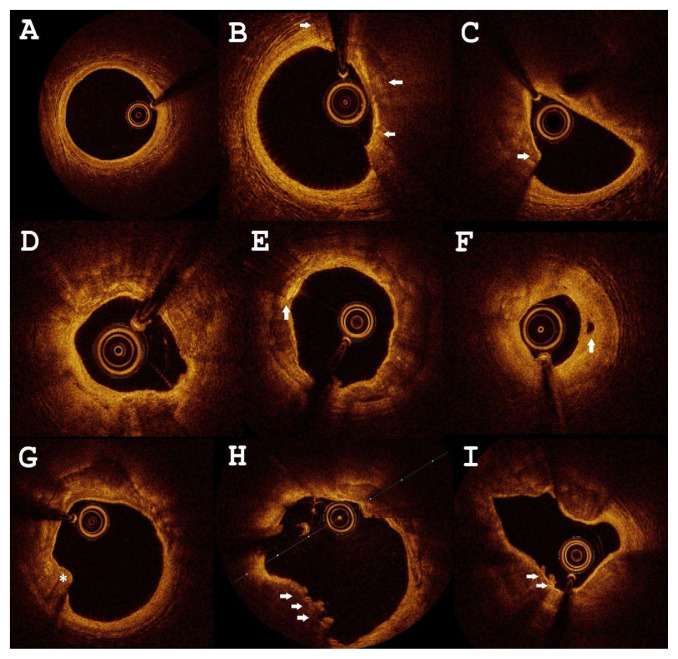
Selected optical coherence tomography findings in a very high-risk subgroup of patients. (**A**) normal healthy vessel; (**B**) thin-cap fibroatheroma (TCFA) with macrophages infiltration (arrows); (**C**) very high-risk plaque defined as TCFA (arrow) phenotype with >180° lipid arc, presence of macrophages and with minimal lumen area <3.5 mm^2^; (**D**) concentric calcification and macrophage accumulation; (**E**) 360° thick calcification with cholesterol crystal (arrow); (**F**) mixed plaque and neovascularization (arrow); (**G**) protruding nodular calcification (asterisk, (**H**) plaque erosion (arrows), (**I**) TCFA and microthrombi (arrows).

Secondary imaging endpoints analyzed on the patient level, associated with plaque vulnerability, involved lipid-rich plaque, spotty calcium, nodular calcification, macrophage accumulation, cholesterol crystal, neovascularization, intraluminal thrombus, and plaque erosion. Representative OCT images of a normal vessel wall and pathological lesions are presented in Figure 1.

#### Statistical analysis

Continuous variables are presented as mean and SDs or medians and IQRs. Nominal variables are shown as counts and percentages. The independent samples *t*-test, Mann-Whitney U Test, or chi-square tests were used to compare differences between the HR and VHR groups, as appropriate. Statistical analyses were conducted with SPSS version 25.0 (IBM Corporation, Armonk, NY). All statistical tests and confidence intervals were 2-sided with a significance level of 0.05.

#### Ethics approval and consent to participate

This study conformed to the provisions of the Declaration of Helsinki and was approved by the ethical committee under No. 10245/23; G-23-22. Informed consent was obtained from all participants involved in this study.

## Results

### Non-invasive screening group (high CV risk patients)

A total of 62 HR patients aged 50.1±12.7 years, 53 % female, were enrolled. Patient characteristics are summarized in Table 1. With regards to ESC guidelines for primary prevention, the target of HbA1c <53 mmol/l and low-density lipoprotein cholesterol (LDL-C) <2.6 mmol/l was achieved in only 32 (52 %) and 27 (44 %) patients, respectively. The criteria of very high cardiovascular risk (VHR) fulfilled 12 of 62 (19.4 %) patients – based on CAC score in 1/62, carotid ultrasonography in 5/62 or both in 6/62 patients.

### Angiographic, functional and OCT assessment (very high-risk patients)

Patients who fulfilled the VHR criteria had a mean age of 64.5 years and 67 % of subjects were female. Patients in the VHR group (n=12), in comparison with the HR group (n=50), were older (64.5 vs. 46.6 years, p=0.001), with longer duration of T1D (36 vs. 25 years, p=0.001), had higher NT-proBNP levels (median of 157.77 vs. 42.8 ng/l, p=0.007), more frequent arterial hypertension (75 % vs. 32 %, p=0.009), lower total cholesterol (4.28 vs. 4.78 mmol/l, p=0.048) and a higher use of statin therapy (83 % vs. 32 %, p=0.002) (Table 2).

The median CAC score in the VHR group was 606.3 (IQR 175.3–1515), and 2.75±1.06 carotid plaques were observed. Coronary angiography showed obstructive coronary artery disease in 42 % (5/12). vFFR of major vessels confirmed hemodynamically significant stenosis in 25 % of patients (3/12), while all three were in left anterior descending (LAD) territory. One patient underwent PCI with a drug-eluting stent (DES) implantation, the second patient was treated conservatively for diffuse atherosclerotic changes, and the third patient had follow-up ICA after twelve months, after which he was indicated for coronary artery bypass grafting.

OCT pullbacks in all three major coronary vessels were successfully acquired in 81 % (29/36) – (LAD – 100 %, LCx – 67 %, RCA – 75 %). On the patient level, thin-cap fibroatheroma was present in 58 % (7/12) and very high-risk plaque in 33 % of patients (4/12) (Table 3). OCT imaging features associated with plaque vulnerability were presented in patients as follows: 92 % (11/12) had lipid-rich plaque, 67 % (8/12) spotty calcium, 33 % (4/12) nodular calcification, 92 % (11/12) macrophage accumulation, 25 % (3/12) cholesterol crystal, 67 % (8/12) neovascularization, 25 % (3/12) thrombus and 50 % (6/12) plaque erosion. Full results of angiographic, functional and OCT qualitative and quantitative measurements are shown in Table 4.

## Discussion

To our knowledge, this is the first OCT study of asymptomatic T1D patients assessing the prevalence of clinically significant coronary lesions in patients with subclinical atherosclerosis determined by the calcium score and carotid ultrasound. The principal findings of this study can be summarized as follows: (1) Majority of T1D patients did not achieve HbA1c and LDL-C primary prevention targets; (2) A large proportion of T1D patients were actually at very high, rather than high, CV risk; (3) The combination of calcium score and carotid ultrasound identified a group of patients with a high prevalence of clinically significant vulnerable plaques in the coronary arteries as defined by the OCT imaging.

In the past, the cardiovascular risk of patients with DM was considered equivalent to that of post-myocardial infarction patients without DM [10]. However, recent data point to a significant heterogeneity in the risk of patients with DM, ranging from low to very high. Only a fifth of people with T2D have a comparable risk to patients with overt cardiovascular disease [11]. Much less is known about the CV risk distribution in patients with T1D. This is partially caused by the lower prevalence of T1D. Furthermore, due to the earlier onset T1D, CVD develops at a younger age. Therefore, an individualized approach based on CVD risk stratification is important. CVD risk estimation affects the intensity of preventive therapy, especially target LDL levels and aspirin indication.

For risk stratification, scores based on traditional cardiovascular risk factors have been used [12]. The current ESC Guidelines on cardiovascular disease prevention in clinical practice recommend classifying T1D patients above 40 years of age according to the same criteria as T2D patients using the SCORE2-Diabetes algorithm. While these scores can stratify patients on the populational level, they often misclassify patients on the individual level. Non-invasive imaging techniques have the potential to improve risk stratification. However, due to the lack of robust evidence, the ESC guidelines do not recommend their routine use [12].

To fill this knowledge gap, we have prospectively evaluated a group of asymptomatic T1D patients without target organ damage and with more than 10 years of T1D duration. We have used CAC>400 and the presence of two or more atherosclerotic plaques in the carotid arteries to define the very high-risk group. CAC score of >400 [13,14,15] or presence of carotid plaques of ≥1.5 mm thickness [16,17] are strong predictors for CVD events. In our study, one in five T1D patients fulfilled the very-high risk criteria.

To confirm the very high risk in patients with subclinical atherosclerosis as defined by CAC and carotid ultrasound, we have used OCT to describe the prevalence of vulnerable plaques in this group. Because most acute coronary syndromes originate from TCFA rupture with subsiding thrombus formation, the presence of TCFA identifies subjects at increased cardiovascular risk. In the COMBINE OCT-FFR study among diabetic patients, FFR-negative lesions containing TCFA were associated with a 5-fold higher rate of adverse events despite the absence of ischemia [2]. Additional features of TCFA as lipid arc >180°, minimum lumen area <3.5 mm^2^ and macrophage accumulation identify patients with 7.5-higher risk of cardiac death and target segment myocardial infarction [9]. In the recent PREVENT trial, preventive PCI of high-risk vulnerable plaques reduces MACE in patients with FFR-negative vulnerable coronary plaques compared to optimal medical therapy alone [2]. On the other hand, in the ISCHEMIA trial with 40 % of participants having diabetes, PCI in patients with moderate or severe ischemia did not decrease cardiovascular risk [18]. This suggests that for prognostication, the search for vulnerable plaque is more clinically relevant than the search for artery stenosis and ischemia. However, vulnerable plaques in the coronary arteries can only be visualized using invasive examination, which is not feasible in routine care. Thus, non-invasive methods pointing to vulnerable plaque presence in the coronary arteries is of clinical importance. In the present study, we show that 58 % of patients with CAC over 400 or at least 2 atherosclerotic plaques in the carotid arteries have a thin cap fibroatheroma, with 1 in 3 of these patients having a very high-risk plaque. Besides plaque rupture, with TCFA as its precursor lesion and the most common cause, other pathological causes of acute coronary syndrome include plaque erosion and protruding nodular calcifications [19]. Moreover, spotty calcification, neovascularization, and cholesterol crystals are also associated with plaque vulnerability [8,20,21]. All of these pathological features were frequently detected on OCT in VHR group. Our study confirms the clinical utility of noninvasive examination of subclinical atherosclerosis in T1D patients.

Real-world data indicates that T1D patients are frequently overlooked in the context of primary CVD prevention. This was corroborated by our study, which found that half of the high-risk asymptomatic patients had inadequate lipid and glycemic control. On the other hand, we observed a high prevalence of vulnerable plaques. Healthcare providers are often reluctant to intensify therapy in asymptomatic patients. However, subclinical atherosclerosis detection may not only help to stratify the CVD risk, but also motivate patients and physicians to comply with guideline-directed targets and thus decrease cardiovascular mortality of T1D patients. The current ESC Guidelines on Cardiovascular Disease Prevention in Clinical Practice recommend to classify T1D patients above 40 years of age according to the same criteria as T2D patients to moderate-, high- and very high-risk patients. For high-risk patients, recommendations include smoking cessation, achieving a target HbA1c below 53 mmol/mol (<7 %), LDL-C levels <2.6 mmol/l, and systolic blood pressure (SBP) between 130–140 mm Hg. However, nearly 20 % of our patients were treated as high-risk instead of very high-risk, potentially leading to suboptimal treatment. For those fulfilling very high-risk criteria, LDL-C<1.4 mmol/l and SBP <130 mm Hg is recommended [4]. The current American College of Cardiology guidelines on primary prevention states common recommendations for T1D and T2D patients as well. Moderate statin therapy is recommended to diabetic patients in 40 to 75 years of age. T1D with a duration of ≥20 years acts as a risk enhancer and high-intensity statin is recommended, although recommendation is not based on any randomized control trial [22]. No aspirin therapy is recommended in the primary prevention of CVD in T1D patients by both guidelines as there is no available evidence of benefit. However, finding of subclinical atherosclerosis in high-risk T1D patients could influence the treatment decision. Analysis of non-diabetic patients showed that a CAC score of ≥100 was associated with favorable risk-benefit estimation for aspirin use [23]. Although findings are not derived from T1D patients, they could be potentially applicable to this population. Whether individualized treatment of T1D patients based on non-invasive imaging leads to cardiovascular risk reduction as compared to the currently used strategy needs to be answered in future randomized studies.

## Study Limitations

The study has several limitations. Firstly, the screened cohort of patients had to consent to the study; therefore, it does not necessarily reflect the general population of T1D patients. Secondly, the study was a single-center study with a relatively small cohort of patients, which may limit the generalizability of the results, which should be confirmed in a larger population. Thirdly, due to ethical considerations, ICA and OCT, despite their low but possible risk of complications, were not performed in patients with low CAC scores or fewer than two carotid plaques, which may have led to the oversight of additional vulnerable plaques. Finally, OCT imaging was performed only in the proximal, technically and anatomically possible, parts of the major coronary arteries, excluding the distal parts, diagonal, or marginal branches.

## Conclusions

Asymptomatic, very high-risk type 1 diabetes mellitus patients, stratified by coronary artery calcium score and carotid ultrasound, frequently present with significant obstructive coronary artery disease, including thin-cap fibroatheroma and high-risk plaque, as diagnosed by OCT.

## Figures and Tables

**Table 1:** Clinical characteristics of T1D patients in the screening phase.

	n=62
*Age, years*	50.05 ± 12.67
*Female gender*	33 (53 %)
*BMI, kg/m* *^2^*	27.24 ± 4.38
*Arterial hypertension*	25 (40 %)
*Hyperlipidemia*	30 (48 %)
*Current smoking*	9 (15 %)
*Obesity*	12 (19 %)
*Diabetes duration, years*	27.35 ± 10.82
*HbA1c*	
*mmol/mol*	56.21 ± 14.72
*%*	7.3 ± 1.3
*No. of patients with HbA1c <53 mmol/mol (7 %)*	32 (52 %)
*Total cholesterol, mmol/l*	4.69 ± 0.79
*LDL cholesterol, mmol/l*	2.64 ± 0.55
*No. of patients with LDL cholesterol <2.6 mmol/l*	27 (44 %)
*HDL cholesterol, mmol/l*	1.61 ± 0.45
*Non* *-* *HDL cholesterol, mmol/l*	3.03 ± 0.9
*Triglycerides, mmol/l*	0.88 (0.68–1.26)
*NT* *-* *proBNP, ng/l*	51.45 (28.38–80.9)
*Acetylsalicylic acid*	4 (6 %)
*Statin*	26 (42 %)
*High* *-* *intensity statin therapy*	1 (2 %)
*Moderate* *-* *intensity statin therapy*	4 (6 %)
*Low* *-* *intensity statin therapy*	21 (34 %)
*Ezetimibe*	6 (10 %)

BMI – body mass index, HDL – high-density lipoprotein, LDL – low-density lipoprotein. NT-proBNP – N-terminal pro B-type natriuretic peptide. High-intensity statin therapy is defined as 40 mg of rosuvastatin or 80 mg of atorvastatin/simvastatin. Moderate-intensity statin therapy is defined as 20 mg of rosuvastatin or 40 mg of atorvastatin/simvastatin. Low-intensity statin therapy is defined as 10 mg of rosuvastatin or 10–20 mg of atorvastatin/simvastatin.

**Table 2:** Comparison of selected clinical characteristics of a high-risk and a very high-risk group.

	High-risk(n=50)	Very high-risk(n=12)	P value
*Age, years*	46.58 ± 11.65	64.5 ± 1.83	**0.001**
*Female gender*	25 (50 %)	8 (67 %)	0.350
*BMI, kg/m* *^2^*	27.04 ± 4.46	28.06 ± 4.06	0.474
*Arterial hypertension*	16 (32 %)	9 (75 %)	**0.009**
*Hyperlipidemia*	20 (40 %)	10 (83 %)	**0.010**
*Current smoking*	9 (18 %)	0 (0 %)	0.185
*Obesity*	9 (75 %)	3 (25 %)	0.686
*Diabetes duration, years*	25.26 ± 9.50	36.08 ± 12.02	**0.001**
*HbA1c, mmol/mol*	55.66 ± 15.79	58.50 ± 9.21	0.553
*No. of patients with HbA1c <53 mmol/mol (7 %)*	29 (58 %)	3 (25 %)	0.055
*Total cholesterol, mmol/l*	4.78 ± 0.78	4.28 ± 0.74	**0.048**
*HDL* *-* *cholesterol, mmol/l*	1.58 ± 0.38	1.76 ± 0.66	0.371
*non* *-* *HDL* *-* *cholesterol, mmol/l*	3.21 ± 0.80	2.31 ± 0.95	**0.001**
*LDL* *-* *cholesterol, mmol/l*	2.67 ± 0.57	2.54 ± 0.47	0.483
*No. of patients with LDL cholesterol <2.6 mmol/l*	21 (42 %)	6 (50 %)	0.749
*Triglycerides, mmol/l*	0.88 (0.7–1.2)	0.75 (0.53–1.59)	0.527
*Apo A1, g/l*	1.71 ±0.31	1.60 ± 0.32	0.273
*Apo B, g/l*	0.79 ± 0.20	0.73 ± 0.13	0.177
*Apo C* *-* *III, mg/l*	146.15 ± 50.67	157.77 ± 70.44	0.512
*Lp(a), nmol/l*	8 (6–19)	15 (6.75–107.3)	0.167
*NT* *-* *proBNP, ng/l*	42.8 (29.1–71.5)	125.3 (45.28–270.8)	**0.007**
*Homocysteine, μmol/l*	8.80 ± 3.02	10.12 ± 2.44	0.168
*Cystatin C, mg/l*	0.96 ±0.10	1.16 ± 0.18	**0.001**
*Acetylsalicylic acid*	(2 %)	3 (25 %)	**0.021**
*Statin*	16 (32 %)	10 (83 %)	**0.002**
*High* *-* *intensity statin therapy*	0 (0 %)	1 (8 %)	0.194
*Moderate* *-* *intensity statin therapy*	2 (4 %)	2 (17 %)	0.166
*Low* *-* *intensity statin therapy*	14 (28 %)	7 (58 %)	**0.086**
*Ezetimibe*	5 (10 %)	1 (8 %)	1.000

BMI – body mass index, HDL – high-density lipoprotein, HR – high-risk group LDL – low-density lipoprotein, Lp(a) – lipoprotein (a), NT-proBNP – N-terminal pro B-type natriuretic peptide. VHR – very high-risk group. High-intensity statin therapy is defined as 40 mg of rosuvastatin or 80 mg of atorvastatin/simvastatin. Moderate-intensity statin therapy is defined as 20 mg of rosuvastatin or 40 mg of atorvastatin/simvastatin. Low-intensity statin therapy is defined as 10 mg of rosuvastatin or 10–20 mg of atorvastatin/simvastatin.

**Table 3:** Angiographic, functional and OCT qualitative and quantitative assessment in the very high-risk group.

	n=12
***Obstructive coronary artery disease***	5 (42 %)

***vFFR*** ***-*** ***positive value ≤0.80***	3 (25 %)

***Optical coherence tomography***	

*Thin-cap fibroatheroma*	7 (58 %)
*Very high-risk plaque*	4 (33 %)
*MLA<3.5 mm^2^*	8 (67 %)
*Lipid-rich plaque*	11 (92 %)
*Maximal lipid plaque arch*	165 ± 100
*Calcification*	8 (67 %)
*Maximal calcification arc*	163 ± 146
*Spotty Calcium*	8 (67 %)
*Nodular calcification*	4 (33 %)
*Macrophage accumulation*	11 (92 %)
*Cholesterol crystal*	3 (25 %)
*Neovascularization*	8 (67 %)
*Thrombus*	3 (25 %)
*Plaque erosion*	6 (50 %)

**Table 4:** Vessel-level angiographic and functional characteristics.

	LAD (n=12)	LCx (n=8)	RCA (n=9)	Overall (n=29)
*Coronary artery calcium score*	322 (90–812)	10 (0–64)	15.85 (0–700)	606.3 (175.3–1515)
*QCA maximal diameter stenosis, %*	30 ± 14	30 ± 15	27 ± 14	29 ± 14
*QCA maximal area stenosis, %*	49 ± 19	49 ± 20	45 ± 21	48 ± 19
*vFFR value*	0.84 ± 0.091	0.95 ± 0.036	0.92 ± 0.046	0.9 ± 0.079
*vFFR value ≤0.80*	3 (25 %)	0 (0 %)	0 (0 %)	3 (10 %)

***Optical coherence tomography characteristics***				

*proximal mean lumen diameter, mm*	3.1 ± 0.48	3.6 ± 0.44	3.3 ± 0.58	3.31 ± 0.53
*distal mean lumen diameter, mm*	2.6 ± 0.52	2.7 ± 0.5	3 ± 0.48	2.77 ± 0.52
*minimal lumen diameter, mm*	1.9 ± 1	1.8 ±0.59	2.2 ±0.54	1.97 ± 0.78
*MLA, mm* *^2^*	3.2 ± 2	3.4 ±1.9	4.9 ± 2.2	3.78 ± 2.03
*MLA <3.5mm* *^2^*	8 (67 %)	5 (63 %)	3 (33 %)	16 (55 %)
*maximal diameter stenosis, %*	34 ± 14	36 ± 13	22 ± 9.8	31 ± 14
*maximal area stenosis, %*	55 ± 19	58 ± 17	39 ± 16	51 ± 19
*Thin* *-* *cap fibroatheroma*	5 (42 %)	4 (50 %)	5 (56 %)	14 (48 %)
*Very high* *-* *risk plaque*	2 (17 %)	3 (38 %)	1 (11 %)	6 (21 %)
*Lipid* *-* *rich plaque*	10 (83 %)	7 (88 %)	6 (67 %)	23 (79 %)
*Maximal lipid plaque arch*	140 ± 86	195 ± 110	113 ± 80	147 ± 94
*Calcification*	8 (67 %)	5 (63 %)	6 (67 %)	19 (66 %)
*Maximal calcification arc*	153 ± 139	30 ± 39	110 ± 132	106 ± 124
*Spotty Calcium*	8 (67 %)	5 (63 %)	6 (67 %)	19 (66 %)
*Nodular calcification*	4 (33 %)	0 (0 %)	2 (22 %)	6 (21 %)
*Macrophage accumulation*	10 (83 %)	7 (88 %)	7 (78 %)	24 (83 %)
*Cholesterol crystal*	2 (17 %)	0 (0 %)	3 (33 %)	5 (17 %)
*Neovascularization*	6 (50 %)	2 (25 %)	5 (56 %)	13 (45 %)
*Thrombus*	3 (25 %)	0 (0 %)	0 (0 %)	3 (10 %)
*Plaque erosion*	5 (42 %)	3 (38 %)	3 (33 %)	11 (38 %)

MLA – minimal lumen area, QCA – quantitative coronary angiography, vFFR – vessel fractional flow reserve.

## Abbreviations

ACS: acute coronary syndrome
CAC: coronary artery calcium
CAD: coronary artery disease
CV: cardiovascular
CVD: cardiovascular disease
DM: diabetes mellitus
FFR: fractional flow reserve
HR: high-risk
ICA: coronary angiography
IMT: intima-media thickness
LAD: left artery descending
LDL-C: low-density lipoprotein cholesterol
LCx: left circumflex artery
MACE: major adverse cardiovascular event
OCT: optical coherence tomography
QCA: quantitative coronary angiography
RCA: right coronary artery
SBP: systolic blood pressure
T1D: type 1 diabetes mellitus
T2D: type 2 diabetes mellitus
TCFA: thin-cap fibroatheroma
vFFR: vessel fractional flow reserve
VHR: very high risk
